# Anti-Inflammatory Effects of Interleukin-19 in Vascular Disease

**DOI:** 10.1155/2012/253583

**Published:** 2012-07-15

**Authors:** Ross N. England, Michael V. Autieri

**Affiliations:** Department of Physiology, Independence Blue Cross Cardiovascular Research Center, Temple University School of Medicine, Philadelphia, PA 19140, USA

## Abstract

Despite aggressive dietary modification, lipid-lowering medications, and other interventional medical therapy, vascular disease continues to be a leading cause of mortality in the western world. It is a significant medical and socioeconomic problem contributing to mortality of multiple diseases including myocardial infarction, stroke, renal failure, and peripheral vascular disease. Morbidity and mortality of vascular disease are expected to worsen with the increasing number of patients with comorbid conditions such as obesity, metabolic syndrome, and diabetes mellitus type 2. Vascular diseases such as atherosclerosis, restenosis, and allograft vasculopathy are recognized to be driven by inflammation, and as such, cytokines which mediate inflammation not only represent important targets of rational therapy, but also can be considered as possible therapeutic modalities themselves. In this paper, we will examine the role of inflammatory cytokines and lymphocyte T_h_1/T_h_2 polarity in vascular inflammation, with a focus on atherosclerotic vascular disease. We will then introduce a recently described T_h_2 interleukin, interleukin-19 (IL-19), as a previously unrecognized mediator of vascular inflammatory disorders. We will review our current understanding of this interleukin in health and disease and present the possibility that IL-19 could represent a potential therapeutic to combat vascular inflammatory disease.

## 1. Inflammation, Cytokines, and Vascular Disease

Inflammation is a ubiquitous pathological process which is central to the development of multiple cardiovascular diseases. Many vascular diseases such as atherosclerosis, restenosis, and transplant vasculopathy are chronic, progressive processes initiated and propagated by local inflammation of large- and medium-sized arteries [1]. This inflammation is mediated by a variety of cell types including macrophage, lymphocyte, endothelial cell (EC), and vascular smooth muscle cell (VSMC). The multiple cell types which participate in vascular inflammation have evolved to produce common cytokines and specific membrane receptors allowing them to transmit their effects into the cell, permitting these diverse cell types to communicate by expression and recognition of multiple pro- and anti-inflammatory cytokines. As such, cytokines and their receptors are the currency of inflammation, and represent attractive targets for therapeutic modalities in numerous vascular inflammatory disorders.

 Synthesis and recognition of cytokines and receptors by both vascular and inflammatory cells allows bidirectional communication between these two systems and demonstrates that, under particular conditions, we can consider vascular cells as an extended participant in the adaptive immune response. Cytokines often act in synergy with other cytokines and frequently share receptor subunits which combine into homodimers or heterodimers with receptors of other cytokines. Cytokines can drive multiple, often simultaneous cellular processes including mitogenesis, development, gene expression, fibrosis, and chemotaxis [2]. This communication initiates a series of receptor-mediated signal transduction cascades including activation of mitogen-activated protein kinases (MAPKs), protein kinase C (PKC), and transcription factors, often including the signal transducer and activator of transcription (STAT) family [3]. Proinflammatory cytokines most often lead to activation of nuclear factor-(NF-*κ*B) which acts as a “master switch” for transcription of numerous genes, the expression of which may be appropriate, as in host defense, or maladaptive, as in chronic vascular disease [4, 5].

 Atherosclerosis is a chronic vascular inflammatory condition mediated by interactions between lymphocytes, macrophage, endothelial, and vascular smooth muscle cells which results in local inflammation of the arterial wall. While an excess of low-density lipoprotein (LDL) is an established risk factor for atherosclerosis, inflammatory mechanisms play an acknowledged role in initiation and propagation of atherogenesis. The inflammatory nature of atherosclerosis has prompted broad investigation into vascular inflammatory processes, and consequently, proinflammatory signaling mechanisms in the vascular wall have been well characterized [6–9]. Less, albeit increasing, interest has been placed on understanding the potentially protective role of anti-inflammatory cell signaling in the vascular wall [10, 11]. Such studies that do exist place a strong emphasis on the role of anti-inflammatory cytokines, for example, interleukin-10 (IL-10), in the immune cells of the plaque. Even less investigation has been carried out on the direct effect of anti-inflammatory cytokines on vascular smooth muscle cells and endothelial cells. Consequently, direct effects of anti-inflammatory cytokines constitute an emerging and promising area of study.

## 2. T_h_1 and T_h_2 Interleukins in Atherosclerosis 

 Interleukins are often classified according to their effects on lymphocyte function or maturation, as T_h_1 (proinflammatory, cytotoxic) which promotes inflammation, and T_h_2 (anti-inflammatory, antibody responses), which generally dampens inflammation [12]. Approximately 10% of the cellular content of human atherosclerotic plaque consists of CD3+ T cells [4]. The overwhelming majority of these are CD4+ helper T cells (T_h_), which recognize epitopes on oxidized LDL [13]. Because atherosclerosis is primarily an inflammatory condition, it is not surprising that T_h_1 interleukins are much more prevalent in human atherosclerotic lesions than the T_h_2 cytokines [9, 12]. T_h_1 cells drive cell-mediated immunity and are characterized by abundant expression of interferon (IFN)-*γ*, IL-12, tumor necrosis factor-*α*, and other proinflammatory cytokines, which are also highly expressed in atherosclerotic lesions. In contrast, T_h_2 cytokines such as IL-4 and IL-10 are far less abundant in human and mouse atherosclerotic lesions. T_h_2 cytokines dampen the inflammatory response through inhibition of proinflammatory genes (including T_h_1 genes) and, conversely, T_h_1 cytokines reduce expression of T_h_2 cytokines. Thus, the very low levels of T_h_2 cytokines detected in atherosclerotic lesions are likely due to the elevated levels of T_h_1 cytokines present. The prevailing hypothesis that atherosclerosis is a T_h_1 disease is supported by studies in which mice lacking IFN-*γ* or the IFN-*γ* receptor, TNF-*α*, or the T_h_1 transcription factor T-bet have reduced atherosclerosis [14–16]. Further supporting this hypothesis, mice lacking STAT6, which is essential for T_h_2 cell differentiation, have increased atherosclerosis [17]. Since so many more proatherogenic cytokines and receptors have been identified and characterized, much greater effort has gone into understanding these cytokines and the potential for inhibition of their expression or activity. Far fewer studies have pursued characterization of anti-inflammatory cytokines in vascular disease. Interleukin-10 is the archetypical T_h_2 interleukin, a potent immune modulator, and the most studied in terms of vascular disease. Several studies have suggested that IL-10 is atheroprotective by several mechanisms including T_h_2 T-cell polarization and attenuation of inflammatory gene expression in inflammatory cells. For example, IL-10 atherosclerotic plaque burden is reduced in IL-10 transgenic mice, and transfer of bone marrow from these mice into LDLR^−/−^ mice reduced atherosclerosis [18]. As expected, atherosclerosis is increased in IL-10^−/−^ mice and IL-10^−/−^/ApoE^−/−^ double knock-out mice. The mechanism is most likely mediated by inflammatory cells, as transfer of IL-10^−/−^ bone marrow to LDLR^−/−^ polarizes the T lymphocyte T_h_2/T_h_1 ratio toward a more anti-inflammatory phenotype [19]. Although considered to be a T_h_2 interleukin, IL-4 does not appear to be antiatherosclerotic, as IL-4^−/−^ mice do not have increased atherosclerosis, and administration of IL-4 into ApoE^−/−^ mice does not reduce development of atherosclerotic lesions [20]. Thus, most studies consider T_h_2 interleukins to be indirectly antiatherogenic by dampening the host immune response, reducing inflammation and lesion formation. Though the number of studies investigating the role of T_h_2 cytokines is dwarfed by those investigating T_h_1, our current understanding suggests that the balance of these two opposing “forces” can dictate outcome of the atherosclerotic lesion. Consequently, investigation of the role of T_h_2 cytokines as potential therapeutics in vascular inflammatory disorders, though understudied as it is, may be considered potentially promising as anti-inflammatory therapy for vascular disorders.

## 3. Discovery and Characterization of Interleukin-19 

Interleukin-19 (IL-19) was first identified and cloned by searching Expressed Sequence Tag (EST) databases for IL-10 homologues [21]. The IL-19 gene is located in chromosome 1q32, in an “IL-10 cluster,” which includes genes for several other IL-10 family members. IL-19 is a member of a subfamily of the IL-10 family of interleukins and is more broadly classified as a class II cytokine, a class which includes the IL-10 family members and the interferons (types I, II, and III) [22–24]. More recently, IL-19 has been classified in a subfamily including IL-19, IL-20, and IL-24, though these subfamily members are recognized by and signal through different combinations of shared receptor chain complexes.

 In its secreted form, human IL-19 is a compact *α*-helical protein composed of 159 amino acids. IL-19 has amino acid identity with IL-10 at 30 residues including 4 cysteines known to be required for correct folding of IL-10 and 41 of the 50 amino acids required for formation of the IL-10 hydrophobic core [21]. The overall IL-19 amino acid sequence shares 20% identity with IL-10, and X-ray crystallography confirms that IL-19 is structurally similar to IL-10 but with key differences [25]. While IL-10 has 6 *α*-helices (A–F), the last of which (F) contributes to the formation of a stable IL-10 homodimer in solution through its insertion into the core of its paired protein, IL-19 has 7 *α*-helices (A–G), the last of which (G) is able to fold back and stabilize IL-19 as a soluble monomer. Further, the homologous region of IL-10 that interacts with the IL-10 receptor chain 1 is far less conserved in IL-19. Together, these properties may explain why IL-19, despite its amino acid identity with IL-10, is not recognized by and cannot signal through the IL-10 receptor complex. 

 IL-19 shares relatively greater structural similarity with fellow subfamily members IL-20 and IL-24, each of which also forms a stable monomer in solution. The genes for these three proteins are found in a gene cluster with IL-10 on chromosome 1 and have been alternately referred to as the “IL-19 subfamily” [22] or the “IL-20 subfamily” [26]. In addition to their structural similarity, interleukins 19, 20, and 24 all signal through receptor complexes containing the IL-20 receptor *β* chain (IL-20R*β*) [27]. All three proteins can signal through the heterodimer formed by IL-20R*α* and IL-20R*β*. IL-20 and IL-24, but not IL-19, can also signal through the receptor formed by IL-22R*α* and IL-20R*β*.

## 4. Expression and Function of IL-19

Expression of IL-19 was first reported by Gallagher et al. [21] in LPS- and GM-CSF-stimulated primary human monocytes, and subsequent early reports on IL-19 focused on its role as a product of immune cells [28]. Among immune cells, IL-19 is primarily expressed by monocytes and, to a lesser extent, by B cells, but some investigators have questioned its role in regulating these cells due to the lack of detectable expression of the IL-20R*α* chain in lymphocytes [28–31]. Notwithstanding the lack of this receptor, effects of IL-19 have been reported in lymphocytes [32, 33], including the notable observation that IL-19 treatment is able to polarize the maturation of human T cells away from the proinflammatory T_h_1 phenotype to the anti-inflammatory T_h_2 phenotype [33, 34]. While expression of IL-20R*α* and IL-20R*β* chains is reported to be cytokineregulated, detailed studies on expression of these peptides in vascular cells or myocytes are lacking. Since its discovery and early characterization, IL-19 expression has been detected in a wide variety of nonimmune human peripheral cell types, including keratinocytes [29], bronchial epithelial cells [35, 36], synovial tissue [37, 38], fetal membranes [39], and vascular endothelial [40] and smooth muscle cells [41] (Table 1). This suggests a functional role for IL-19 distinct from T_h_1/T_h_2 polarization. Paradoxically, IL-19 seems to exert both proinflammatory and anti-inflammatory properties in a manner contextually governed by tissue-specific and disease-specific factors. The myriad roles of IL-19 in noncardiovascular tissues and diseases are of qualified interest to the scope of this paper as other roles of IL-19 could affect its efficacy and desirability as a therapeutic modality in vascular disease. The multiple effects of IL-19 have been well reviewed in the past [22] and will be briefly presented here.

 A putative role for IL-19 has been put forth in the development of psoriasis, a chronic inflammatory skin condition characterized by increased proliferation of keratinocytes leading to the development of plaque-like epidermal lesions. Expression of IL-19, IL20R*α*, and IL20R*β* can be detected in psoriatic lesions [29, 44, 47, 56, 57], and treatment of psoriasis reduces expression of IL-19 [47, 56]. Current findings suggest a possible feedback loop whereby IL-19 promotes expression of keratinocyte growth factor (KGF) in CD8+ T cells which, in turn, induces increased expression of IL-19 from keratinocytes [44]. However, while IL-20 transgenic mice are reported to have a psoriatic phenotype, IL-19 mice exhibit no such pathology. A causal role for IL-19 in psoriasis has not been well established, though the data support a stronger implication for the IL-19 family member IL-20 in this disease.

T_h_2 cytokines are involved in the pathogenesis of a number of diseases, most notably asthma [58–60], a chronic inflammatory airway disease resulting in bronchospasm and consequent reversible airway obstruction. As expected, given the T_h_2 nature of the disease, IL-19 has a demonstrable, though yet unclear, role in the development of asthma. IL-19 expression is increased in the lungs of mice exposed to allergens [42]. Serum IL-19 levels are increased in children with asthma when compared with normal children [42], and airway epithelial cells of asthma patients exhibit increased IL-19 expression [36]. IL-19 expression in airway cells can be modulated by adenosine receptors [48], which play a role in asthma-related cell signaling. 

 Recent scientific interest in IL-19 has prompted investigators to pursue exploration of IL-19 involvement in various other diseases and tissue types. IL-19 has been indicated as potentially protective against gut inflammation [50, 61], representing the potential for therapeutic use in inflammatory bowel disease. IL-19 has a suggested role in promoting the development of endotoxic (“septic”) shock [49] as well as rheumatoid arthritis [38, 54]. Recent work demonstrated IL-19 expression in numerous neoplastic cell types, including cells of squamous cell carcinoma of the oral cavity, in which IL-19 promoted proliferation [52]. IL-19 also promoted proliferation and migration of breast cancer cells, and high IL-19 expression was associated with poor outcomes in breast cancer patients [53]. 

## 5. Expression of Interleukin-19 in Vascular Disease

Vascular expression of IL-19 was first identified in 2005 through cDNA microarray analysis of cultured human vascular smooth muscle cells treated with inflammatory stimuli [62]. This was unexpected as IL-19 expression had previously been thought to be restricted to leukocytes [21, 22, 34, 63]. Induction of IL-19 expression in vascular cells was further characterized, and western blot analysis of cultured human VSMC demonstrated that IL-19, while not expressed in quiescent (unstimulated) controls, can be induced in VSMC treated with inflammatory stimuli including fetal bovine serum (FBS), T-cell-conditioned media (TCM), IFN-*γ*, platelet-derived growth factor (PDGF), and TNF-*α* [41]. Analysis of endothelial cells produced similar results, showing that microvascular EC (mEC), coronary artery EC (CaEC), and human vascular EC (HVEC) can all be stimulated to express IL-19 by FBS, basic fibroblast growth factor (bFGF), vascular endothelial growth factor (VEGF), and oxidized LDL (ox-LDL) [40]. In contrast, IL-10 expression could not be detected in VSMC at the mRNA or protein level [41]. In histological analysis of human coronary arteries, IL-19 expression was undetectable in sections from normal arteries, but was highly expressed in EC [40], neointimal and medial VSMC [41], and CD45+ leukocytes in coronary arteries with allograft vasculopathy, a chronic vascular inflammatory syndrome. Similarly, both IL-20 and its receptor subunits are expressed in macrophage and EC in atherosclerotic plaque and are induced in these cultured cells when stimulated with inflammatory factors. IL-20 is not expressed in VSMC. In contrast, IL-19 is detected in VSMC [41], EC [40], and CD45+ leukocytes [41] in atherosclerotic plaque in aortic arch of ApoE^−/−^mice, but not aortic arch of wild-type mice, further suggesting that IL-19 is only expressed in response to vascular injury. 

 In another connection to vascular disease, two reports indicate that serum concentrations of IL-19 are increased in patients undergoing coronary artery bypass graft (CABG) surgery with cardiopulmonary bypass [45, 64], and that the increased IL-19 levels contribute to the cell-mediated immune suppression frequently observed in these patients [45].

## 6. Pleiotropic Effects of IL-19 on Vascular Cells

**Figure 1:**
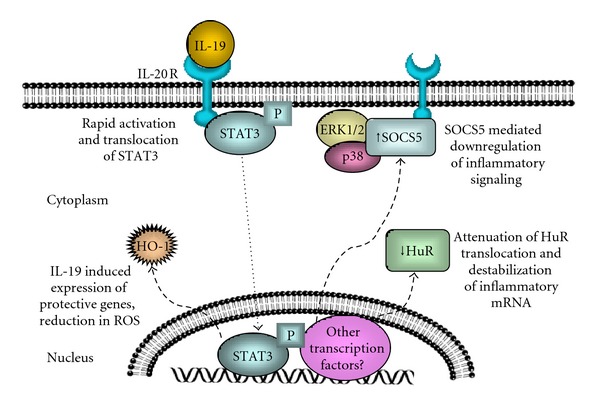
Proposed pleiotropic mechanisms of IL-19 effects in vascular smooth muscle cells. Several mechanisms occurring independently or concurrently may be responsible for IL-19 anti-inflammatory effects on VSMC. These include STAT3-mediated SOCS5 expression, which dampens early activation events such as signal transduction. The second is a down-regulation of HuR abundance and cytoplasmic translocation, resulting in attenuation of proliferative and inflammatory gene expression. The third is expression of HO-1, with subsequent protective effects attributed to HO-1 such as reduction of intracellular ROS. These mechanisms are not mutually exclusive, so varying degrees or combinations of each are likely.

In vascular disease, where the participating cell types are primarily immune cells and vascular cells, IL-19 exerts a pronounced anti-inflammatory effect (Table 2). IL-19 has antiproliferative effects on the NIH:OVCAR-3 ovarian carcinoma cell line [51], and IL-10 has antiproliferative effects in vascular cells [65], suggesting that vascular expression of IL-19 in response to injury might represent a novel autocrine or paracrine mechanism for attenuation and regulation of VSMC proliferation. Several experiments were carried out to test this hypothesis and have uncovered multiple potential mechanisms for these effects (summarized in Figure 1). Treatment of cultured VSMC with recombinant IL-19 or with adenoviral expression of IL-19 decreased VSMC proliferation compared to controls in a concentration-dependent manner [41]. *In vivo* experiments in rats recapitulated this antiproliferative effect, demonstrating that adenoviral delivery of IL-19 to balloon angioplasty-injured rat carotid arteries decreased neointima formation and number of proliferating (Ki-67-positive) VSMCs in this tissue. IL-19 treatment of VSMCs evoked a rapid and transient activation of STAT3 as measured by both phosphorylation and nuclear translocation. IL-19 was shown to rapidly increase expression of suppressor of cytokine signaling 5 (SOCS5), an STAT-responsive gene, at both the mRNA and protein levels. IL-19-induced SOCS5 expression was dependent on STAT3 [41]. There are six SOCS family members which function to suppress cytokine signaling by binding to phosphorylated tyrosine residues on cytokine receptors and cytoplasmic signaling intermediates and targeting them for E3-ubiquitin ligase-mediated degradation. SOCS-mediated signaling inhibition is a strategy employed by numerous cytokines [66]. In VSMC, IL-19 can reduce fetal bovine serum-induced activation of the p44/42 and p38 MAPKs, both mediators of inflammation. IL-19-induced SOCS5 binds the p44/42 and p38 MAPKs, providing at least one probable mechanism for these effects [41]. This indicates that IL-19 can reduce VSMC activation by inhibition of signal transduction. In addition to IL-19, this work also implicates SOCS5 as an important mediator of anti-inflammatory signal transduction.

 IL-19 can decrease FBS-mediated induction of protein and mRNA abundance of proliferative and proinflammatory genes in VSMC, including Cyclin D1, cyclooxygenase-2 (COX-2), IL-1*β*, and IL-8 [68]. Interestingly, this inhibition was selective, and other important regulatory proteins such as proliferating cell nuclear antigen (PCNA), Rac1, and others were not sensitive to IL-19. Other T_h_2 interleukins also reduce inflammatory cytokine expression, and IL-10 in particular reduces inflammatory responses in varied cell types by inhibition of activation of the transcription factor NF-*κ*B. In contrast to IL-10, IL-19 did not inhibit NF-*κ*B activation, as determined by I*κ*B degradation and p65 subunit phosphorylation in both VSMC [68] and EC [67]. This surprising finding suggested that IL-19 decreases abundance of inflammatory and proliferative genes in an NF-*κ*B-independent mechanism and prompted a search for other possible mechanisms whereby IL-19 could decrease inflammatory gene abundance without affecting their transcription. Notably, many proinflammatory genes, including the genes affected by IL-19, are targeted for preferential degradation by cis-acting AU-rich elements (AREs) in their 3′ untranslated regions [71]. Two proteins, human R antigen (HuR) and AU-rich RNA-binding factor-1 (AUF-1), have been shown to regulate ARE-bearing transcripts by binding to ARE and modifying their mRNA stability [72], with HuR promoting increased mRNA stability and AUF-1 promoting decreased stability. The half-lives of ARE-bearing transcripts may be regulated in a “yin-yang” fashion through competitive binding of HuR and AUF-1. The ability of HuR to stabilize mRNA corresponds with its translocation from a predominately nuclear location into the cytoplasm [72]. Prompted by the observation that IL-19-inhibited transcripts bear AREs, and that transcripts lacking AREs (e.g., PCNA) are unaffected by IL-19, the effects of IL-19 on HuR, AUF-1, and mRNA stability were explored. It was found that IL-19 reduces HuR translocation in both FBS-stimulated VSMC [68] and TNF-*α*-stimulated human coronary artery EC [67]. AUF-1 abundance and translocation were not affected by IL-19. As expected, IL-19 reduced stability of inflammatory and proliferative mRNA transcripts which contained ARE in both VSMC and EC, as measured using qRT-PCR and with the transcription inhibitor actinomycin D, but failed to affect stability of mRNA lacking AREs. This effect on stability was able to be recapitulated using HuR siRNA [67, 68]. Taken together, these observations suggested that IL-19 signaling in vascular cells is permissive of NF-*κ*B-mediated increases in inflammatory and proliferative gene *transcription*; however, IL-19 produces a *posttranscriptional* decrease in the abundance of these transcripts through inhibition of HuR translocation, thereby mediating a decrease in transcript stability. This represents a second mechanism, in addition to IL-19-induced SOCS5 expression, through which IL-19 may exert its anti-inflammatory effects on vascular cells.

 IL-19 has been shown to have a direct effect on VSMC motility. IL-19 inhibited cultured VSMC remigration into a scratch wound and also inhibited PDGF-induced migration in a Boyden chamber [69]. Molecular analysis revealed that IL-19 inhibits activation of cellular motility proteins, including myosin light chain (MLC), cofilin, Hsp70, and the monomeric G proteins Rac1 and RhoA. The precise molecular mechanism(s) of IL-19 decrease in activation of these important proteins remains to be elucidated.

 In contrast to its documented antiproliferative effects in VSMC, IL-19 exhibits proliferative, promigratory, and proangiogenic effects in vascular EC. Recombinant IL-19 treatment of EC *in vitro* results in activation of STAT3, Rac1, and MAPK p44/42 with consequent increases in EC proliferation, spreading, and migration. Confirming its proangiogenic potential, IL-19 promotes formation of endothelial cell tubes in isolated cultured mouse aortic rings and promotes formation of nascent blood vessels in subcutaneous gel plugs in mice [40]. These functions are independent of bFGF and VEGF expression and are IL-19-specific, as specific antibody to IL-20 receptor significantly reduces IL-19-driven EC migration [37]. The molecular basis of these intriguing and unexpected observations should uncover interesting distinctions between EC and VSMC processing of anti-inflammatory signals.

 Heme oxygenase-1 (HO-1) has powerful anti-inflammatory and antiapoptotic effects and protects against vascular inflammation through multiple mechanisms including decreasing monocyte arterial transmigration, decreasing VSMC proliferation, and acting as a potent antioxidant [73, 74]. HO-1 is induced primarily at the transcriptional level by many proinflammatory mediators including cytokines, oxidative stress, and some growth factors [75]. IL-19 can induce expression of HO-1 mRNA and protein in cultured VSMC, but not EC [70], again, another interesting distinction in IL-19 cell-specific effects. Consistent with this finding, IL-19 can reduce peroxide-induced apoptosis and growth-factor-induced reactive oxygen species (ROS) accumulation in VSMC. This reduction in ROS was abrogated when VSMCs were transfected with HO-1-specific siRNA prior to IL-19 treatment. *In vivo*, IL-19 can reduce TNF-*α*-induced ROS accumulation in murine coronary arteries [70]. While it has been shown that IL-10 can induce HO-1 in monocyte/macrophages [76], induction of HO-1 in vascular cells by any anti-inflammatory cytokine or T_h_2 interleukin had not been reported. This provides a third potential molecular mechanism whereby IL-19 can reduce vascular inflammation and implicates IL-19 as a potential link between two powerful and protective systems, anti-inflammation and reduction of ROS. 

 In unpublished experiments, LDLR^−/−^ mice fed an atherogenic diet and injected with as little as 1.0 ng/g/day of recombinant IL-19 demonstrated significantly less atherosclerotic plaque lesion area in the aortic arch compared with PBS-injected control mice [77]. These mice have decreased macrophage infiltrate into the atherosclerotic lesion. Interestingly, based on serum cytokine analysis, preliminary data suggest that these mice do not have an altered T_h_1 /T_h_2 balance. This is in contrast to IL-20, which is proatherosclerotic [78]. Further, weight gain as well as serum cholesterol and triglyceride levels is identical in IL-19-treated mice compared with PBS controls. This is an important distinction for IL-19, as several studies have shown an association between T_h_1 /T_h_2 balance with hypercholesterolemia [79, 80]. Together, these preliminary, but provocative, data suggest that IL-19 can decrease atherosclerosis in susceptible mice while neither affecting T_h_1 /T_h_2 balance nor suppressing serum lipid levels and place emphasis on vascular cells as the primary targets for IL-19. Future studies are necessary to determine the precise molecular and cellular mechanisms for IL-19-mediated decreases in vascular disease.

## 7. Summary, Conclusions, and Future Perspectives

The roles of cytokines in development of vascular inflammatory diseases such as atherosclerosis, restenosis, and coronary artery transplant vasculopathy are very complex. It is clear however that the functions of putative anti-inflammatory cytokines in these disease processes hold potential as therapeutics and require further study to characterize their precise mechanism(s) of action. Interleukin-19 is rather unique among interleukins, and its expression by resident vascular cells may represent an autoregulatory, autocrine, or paracrine mechanism to promote resolution of the vascular response to inflammatory insult. IL-19 is not detectible in naïve artery, but is induced in response to vascular injury and inflammation. Similarly, IL-19 is expressed in EC and VSMC when stimulated with inflammatory stimuli, and its addition to these cell types imparts anti-inflammatory effects, with decrease in ROS abundance, migration, proliferation, and expression of inflammatory genes. Function of IL-19 outside of the immune system implies that resident vascular cells may take on a T_h_2 phenotype, and the pleiotropic mechanisms of IL-19 in vascular cells suggest that IL-19 may be a valuable anti-inflammatory therapeutic modality in acute vascular injury such as balloon angioplasty as well as more chronic vascular inflammatory diseases such as allograft vasculopathy and atherosclerosis.

## Figures and Tables

**Table 1:** Effects of IL-19 in nonvascular cell types.

Tissue type	Effect	Species	Source
Immune cells	T_h_2 response in T cells	h, m	[33, 34, 42]
	Inhibits IFN-*γ* production in T cells	h	[34]
	Induces IL-4 and IL-13 production in T cells	h	[34]
	Induces IL-10 production in monocytes	h	[43]
	Autoinduces IL-19 expression in PBMC; dendritic cells	h	[43]
	Induce KGF expression in CD8+ T cells	h	[44]
	Suppress cell-mediated immunity in postbypass patients	h	[45]
	Induced production of IL-6; TNF-*α* in monocytes	m	[46]
	Induced ROS production and apoptosis in monocytes	m	[46]

Skin cells	Expressed in keratinocytes in psoriatic skin	h	[29, 47]
	STAT3 phosphorylation in HaCat keratinocyte cell line	h	[33]

Airway epithelium	HBEC-produced IL-19 induces TNF-*α* production in THP-1 monocyte line	h	[48]
	Induced apoptosis in lung epithelium cells	h	[49]

Colon epithelium	IL-19 expression is protective against dextran sucrose sodium-induced colitis	m	[50]

Cancer cells	Inhibit proliferation in NIH:OVCAR3 (ovarian carcinoma) cells	h	[51]
	Increase proliferation in oral squamous cell carcinoma cells	h	[52]
	Increase proliferation in breast cancer cells	h, m	[53]
	Induced IL-1*β*, IL-6, TGF-*β*, MMP-2, -9, and CXCR4 in 4T1 breast cancer cells *in vitro *	m	[53]
	Induced fibronectin expression in 4T1 breast cancer cells *in vitro *	m	[53]

Fetal membranes	Induce IL-6 production	h	[39]
	Inhibit LPS-induced TNF-*α* production	h	[39]

Liver	Induced ROS production in Huh-7 cell line	h	[49]

Synovial fluid	Inhibits apoptosis in RASC	h	[37]
	Activates STAT3 and induces IL-6 production in RASC	h	[37]
	Induces TNF-*α*, IL-1*β*, IL-6, and RANKL in collagen-induced arthritis synovial fibroblasts	r	[54]

Nasal fibroblast	Inhibits IL-4-induced eotaxin expression by SOCS1-dependent mechanism	h	[55]

Abbreviations: HBEC: human bronchial epithelial cell, RASC: rat arthritis synovial cell, h: human, m: mouse, r: rat.

**Table 2:** Effects of IL-19 on resident vascular cells.

Tissue type	Effect	Species	Source
EC	Autoinduces IL-19 expression	h	[40]
	Activates STAT3, Rac1, and MAPK p44/42	h	[40]
	Increases EC proliferation	h	[40]
	Increases EC spreading, and migration	h	[40]
	Proangiogenic (increase tube, microvessel formation)	m	[40]
	Inhibits HuR nucleocytoplasmic translocation	h	[67]

VSMC	Inhibits proliferation, hyperplasia	h, r	[41]
	Autoinduces IL-19 expression	h	[41]
	STAT3 phosphorylation, translocation	h	[41]
	Increases SOCS5 expression	h	[41]
	Inhibits MAPKs (p44/42, p38)	h	[41]
	Decreases inflammatory, proliferative proteins, and mRNAs	h	[68]
	Decreases HuR protein abundance	h	[68]
	Inhibits HuR nucleocytoplasmic translocation	h	[68]
	Decreases ARE-bearing mRNA stability	h	[68]
	Inhibits PKC*α* activation	h	[68]
	Inhibits migration, spreading	h	[69]
	Inhibits activation of MLC, cofilin, Hsp70, Rac1, and RhoA	h	[69]
	STAT3-dependent increase heme oxygenase-1 expression	h	[70]
	Decreases ROS *in vitro* and *in vivo *	h, m	[70]
	Inhibits apoptosis	h	[70]

Abbreviations: h: human, m: mouse, r: rat.

## References

[1] Ross R (1999). Atherosclerosis—an inflammatory disease. *New England Journal of Medicine*.

[2] Tedgui A, Mallat Z (2006). Cytokines in atherosclerosis: pathogenic and regulatory pathways. *Physiological Reviews*.

[3] Paukku K, Silvennoinen O (2004). STATs as critical mediators of signal transduction and transcription: lessons learned from STAT5. *Cytokine and Growth Factor Reviews*.

[4] Hansson GK, Libby P (2006). The immune response in atherosclerosis: a double-edged sword. *Nature Reviews Immunology*.

[5] Brand K, Page S, Walli AK, Neumeier D, Baeuerle PA (1997). Role of nuclear factor-*κ*B in atherogenesis. *Experimental Physiology*.

[6] Ross R (1993). The pathogenesis of atherosclerosis: a perspective for the 1990s. *Nature*.

[7] Raines EW, Ferri N (2005). Cytokines affecting endothelial and smooth muscle cells in vascular disease. *Journal of Lipid Research*.

[8] Singer CA, Salinthone S, Baker KJ, Gerthoffer WT (2004). Synthesis of immune modulators by smooth muscles. *BioEssays*.

[9] Von der Thüsen JH, Kuiper J, Van Berkel TJC, Biessen EAL (2003). Interleukins in atherosclerosis: molecular pathways and therapeutic potential. *Pharmacological Reviews*.

[10] Tedgui A, Mallat Z (2001). Anti-inflammatory mechanisms in the vascular wall. *Circulation Research*.

[11] Cuneo AA, Autieri MV (2009). Expression and function of anti-inflammatory interleukins: the other side of the vascular response to injury. *Current Vascular Pharmacology*.

[12] Frostegård J, Ulfgren AK, Nyberg P (1999). Cytokine expression in advanced human atherosclerotic plaques: dominance of pro-inflammatory (Th1) and macrophage-stimulating cytokines. *Atherosclerosis*.

[13] Stemme S, Faber B, Holm J, Wiklund O, Witztum JL, Hansson GK (1995). T lymphocytes from human atherosclerotic plaques recognize oxidized low density lipoprotein. *Proceedings of the National Academy of Sciences of the United States of America*.

[14] Gupta S, Pablo AM, Jiang XC, Wang N, Tall AR, Schindler C (1997). IFN-*γ*, potentiates atherosclerosis in ApoE knock-out mice. *Journal of Clinical Investigation*.

[15] Elhage R, Jawien J, Rudling M (2003). Reduced atherosclerosis in interleukin-18 deficient apolipoprotein E-knockout mice. *Cardiovascular Research*.

[16] Buono C, Binder CJ, Stavrakis G, Witztum JL, Glimcher LH, Lichtman AH (2005). T-bet deficiency reduces atherosclerosis and alters plaque antigen-specific immune responses. *Proceedings of the National Academy of Sciences of the United States of America*.

[17] Huber SA, Sakkinen P, David C, Newell MK, Tracy RP (2001). T helper-cell phenotype regulates atherosclerosis in mice under conditions of mild hypercholesterolemia. *Circulation*.

[18] Pinderski LJ, Fischbein MP, Subbanagounder G (2002). Overexpression of interleukin-10 by activated T lymphocytes inhibits atherosclerosis in LDL receptor-deficient mice by altering lymphocyte and macrophage phenotypes. *Circulation Research*.

[19] Mallat Z, Besnard S, Duriez M (1999). Protective role of interleukin-10 in atherosclerosis.. *Circulation research*.

[20] King VL, Cassis LA, Daugherty A (2007). Interleukin-4 does not influence development of hypercholesterolemia or angiotensin II-induced atherosclerotic lesions in mice. *American Journal of Pathology*.

[21] Gallagher G, Dickensheets H, Eskdale J (2000). Cloning, expression and initial characterisation of interleukin-19 (IL-19), a novel homologue of human interleukin-10 (IL-10). *Genes and Immunity*.

[22] Gallagher G (2010). Interleukin-19: multiple roles in immune regulation and disease. *Cytokine and Growth Factor Reviews*.

[23] Sabat R (2010). IL-10 family of cytokines. *Cytokine and Growth Factor Reviews*.

[24] Ouyang W, Rutz S, Crellin NK, Valdez PA, Hymowitz SG (2011). Regulation and functions of the IL-10 family of cytokines in inflammation and disease. *Annual Review of Immunology*.

[25] Chang C, Magracheva E, Kozlov S (2003). Crystal structure of interleukin-19 defines a new subfamily of helical cytokines. *Journal of Biological Chemistry*.

[26] Sa SM, Valdez PA, Wu J (2007). The effects of IL-20 subfamily cytokines on reconstituted human epidermis suggest potential roles in cutaneous innate defense and pathogenic adaptive immunity in psoriasis. *Journal of Immunology*.

[27] Dumoutier L, Leemans C, Lejeune D, Kotenko SV, Renauld JC (2001). Cutting edge: STAT activation by IL-19, IL-20 and mda-7 through IL-20 receptor complexes of two types. *Journal of Immunology*.

[28] Wolk K, Kunz S, Asadullah K, Sabat R (2002). Cutting edge: immune cells as sources and targets of the IL-10 family members?. *Journal of Immunology*.

[29] Kunz S, Wolk K, Witte E (2006). Interleukin (IL)-19, IL-20 and IL-24 are produced by and act on keratinocytes and are distinct from classical ILs. *Experimental Dermatology*.

[30] Wolk K, Witte K, Witte E (2008). Maturing dendritic cells are an important source of IL-29 and IL-20 that may cooperatively increase the innate immunity of keratinocytes. *Journal of Leukocyte Biology*.

[31] Nagalakshmi ML, Murphy E, McClanahan T, De Waal Malefyt R (2004). Expression patterns of IL-10 ligand and receptor gene families provide leads for biological characterization. *International Immunopharmacology*.

[32] Wahl C, Müller W, Leithäuser F (2009). IL-20 receptor 2 signaling down-regulates antigen-specific T cell responses. *Journal of Immunology*.

[33] Gallagher G, Eskdale J, Jordan W (2004). Human interleukin-19 and its receptor: a potential role in the induction of Th2 responses. *International Immunopharmacology*.

[34] Oral HB, Kotenko SV, Yilmaz M (2006). Regulation of T cells and cytokines by the interleukin-10 (IL-10)-family cytokines IL-19, IL-20, IL-22, IL-24 and IL-26. *European Journal of Immunology*.

[35] Aujla SJ, Chan YR, Zheng M (2008). IL-22 mediates mucosal host defense against Gram-negative bacterial pneumonia. *Nature Medicine*.

[36] Huang F, Wachi S, Thai P (2008). Potentiation of IL-19 expression in airway epithelia by IL-17A and IL-4/IL-13: important implications in asthma. *Journal of Allergy and Clinical Immunology*.

[37] Sakurai N, Kuroiwa T, Ikeuchi H (2008). Expression of IL-19 and its receptors in RA: potential role for synovial hyperplasia formation. *Rheumatology*.

[38] Alanärä T, Karstila K, Moilanen T, Silvennoinen O, Isomäki P (2010). Expression of IL-10 family cytokines in rheumatoid arthritis: elevated levels of IL-19 in the joints. *Scandinavian Journal of Rheumatology*.

[39] Menon R, Ismail L, Ismail D, Merialdi M, Lombardi SJ, Fortunato SJ (2006). Human fetal membrane expression of IL-19 and IL-20 and its differential effect on inflammatory cytokine production. *Journal of Maternal-Fetal and Neonatal Medicine*.

[40] Jain S, Gabunia K, Kelemen SE, Panetti TS, Autieri MV (2011). The anti-inflammatory cytokine interleukin 19 is expressed by and angiogenic for human endothelial cells. *Arteriosclerosis, Thrombosis, and Vascular Biology*.

[41] Tian Y, Sommerville LJ, Cuneo A, Kelemen SE, Autieri MV (2008). Expression and suppressive effects of interleukin-19 on vascular smooth muscle cell pathophysiology and development of intimal hyperplasia. *American Journal of Pathology*.

[42] Liao S-C, Cheng Y-C, Wang Y-C (2004). IL-19 induced Th2 cytokines and was up-regulated in asthma patients. *Journal of Immunology*.

[43] Jordan WJ, Eskdale J, Boniotto M (2005). Human IL-19 regulates immunity through auto-induction of IL-19 and production of IL-10. *European Journal of Immunology*.

[44] Li H-H, Lin Y-C, Chen PJ (2005). Interleukin-19 upregulates keratinocyte growth factor and is associated with psoriasis. *British Journal of Dermatology*.

[45] Yeh C-H, Cheng B-C, Hsu C-C (2011). Induced interleukin-19 contributes to cell-mediated immunosuppression in patients undergoing coronary artery bypass grafting with cardiopulmonary bypass. *Annals of Thoracic Surgery*.

[46] Liao Y-C, Liang W-G, Chen F-W, Hsu J-H, Yang J-J, Chang M-S (2002). IL-19 induces production of IL-6 and TNF-*α* and results in cell apoptosis through TNF-*α*. *Journal of Immunology*.

[47] Ghoreschi K, Thomas P, Breit S (2003). Interleukin-4 therapy of psoriasis induces Th2 responses and improves human autoimmune disease. *Nature Medicine*.

[48] Zhong H, Wu Y, Belardinelli L, Zeng D (2006). A2B adenosine induce IL-19 from bronchial epithelial cells, resulting in TNF-*α* increase. *American Journal of Respiratory Cell and Molecular Biology*.

[49] Hsing C-H, Chiu C-J, Chang L-Y, Hsu CC, Chang MS (2008). IL-19 is involved in the pathogenesis of endotoxic shock. *Shock*.

[50] Azuma YT, Matsuo Y, Kuwamura M (2010). Interleukin-19 protects mice from innate-mediated colonic inflammation. *Inflammatory Bowel Diseases*.

[51] Parrish-Novak J, Xu W, Brender T (2002). Interleukins 19, 20, and 24 signal through two distinct receptor complexes: differences in receptor-ligand interactions mediate unique biological functions. *Journal of Biological Chemistry*.

[52] Hsing C-H, Li H-H, Hsu YH (2008). The distribution of interleukin-19 in healthy and neoplastic tissue. *Cytokine*.

[53] Hsing C-H, Cheng H-C, Hsu Y-H (2012). Upregulated IL-19 in breast cancer promotes tumor progression and affects clinical outcome. *Clinical Cancer Research*.

[54] Hsu Y-H, Hsieh P-P, Chang M-S (2012). Interleukin-19 blockade attenuates collagen-induced arthritis in rats. *Rheumatology*.

[55] Higashino M, Takabayashi T, Takahashi N (2011). Interleukin-19 downregulates interleukin-4-induced eotaxin production in human nasal fibroblasts. *Allergology International*.

[56] Rømer J, Hasselager E, Nørby PL, Steiniche T, Clausen JT, Kragballe K (2003). Epidermal overexpression of interleukin-19 and -20 mRNa in psoriatic skin disappears after short-term treatment with cyclosporine A or calcipotriol. *Journal of Investigative Dermatology*.

[57] Otkjaer K, Kragballe K, Funding AT (2005). The dynamics of gene expression of interleukin-19 and interleukin-20 and their receptors in psoriasis. *British Journal of Dermatology*.

[58] Walker C, Kaegi MK, Braun P, Blaser K (1991). Activated T cells and eosinophilia in bronchoalveolar lavages from subjects with asthma correlated with disease severity. *Journal of Allergy and Clinical Immunology*.

[59] Walker C, Bode E, Boer L, Hansel TT, Blaser K, Virchow JC (1992). Allergic and nonallergic asthmatics have distinct patterns of T-cell activation and cytokine production in peripheral blood and bronchoalveolar lavage. *American Review of Respiratory Disease*.

[60] Robinson DS, Hamid Q, Ying S (1992). Predominant T(H2)-like bronchoalveolar T-lymphocyte population in atopic asthma. *New England Journal of Medicine*.

[61] Azuma Y-T, Matsuo Y, Nakajima H (2011). Interleukin-19 is a negative regulator of innate immunity and critical for colonic protection. *Journal of Pharmacological Sciences*.

[62] Kelemen SF, Eisen HJ, Autieri MV (2005). Expression of the FAST-1 transcription factor in coronary artery transplant vasculopathy and activated vascular smooth muscle cells. *Journal of Heart and Lung Transplantation*.

[63] Sabat R, Wallace E, Endesfelder S, Wolk K (2007). IL-19 and IL-20: two novel cytokines with importance in inflammatory diseases. *Expert Opinion on Therapeutic Targets*.

[64] Hsing CH, Hsieh MY, Chen WY, Cheung So E, Cheng BC, Chang MS (2006). Induction of interleukin-19 and interleukin-22 after cardiac surgery with cardiopulmonary bypass. *Annals of Thoracic Surgery*.

[65] Pinderski Oslund LJ, Hedrick CC, Olvera T (1999). Interleukin-10 blocks atherosclerotic events in vitro and in vivo. *Arteriosclerosis, Thrombosis, and Vascular Biology*.

[66] Fujimoto M, Naka T (2003). Regulation of cytokine signaling by SOCS family molecules. *Trends in Immunology*.

[67] England RN, Kelemen SE, Ellison SP, Preston K, Scalia R, Autieri MV IL-19 reduces endothelial cell adhesion molecule abundance and leukocyte-endothelial cell interaction by a decrease in HuR activity and mRNA stabilit.

[68] Cuneo AA, Herrick D, Autieri MV (2010). Il-19 reduces VSMC activation by regulation of mRNA regulatory factor HuR and reduction of mRNA stability. *Journal of Molecular and Cellular Cardiology*.

[69] Gabunia K, Jain S, England RN, Autieri MV (2011). Anti-inflammatory cytokine interleukin-19 inhibits smooth muscle cell migration and activation of cytoskeletal regulators of VSMC motility. *American Journal of Physiology, Cell Physiology*.

[70] Gabunia K, Ellison SP, Singh H (2012). Interleukin-19 (IL-19) induces Heme Oxygenase-1 (HO-1) expression and decreases reactive oxygen species in human vascular smooth muscle cells. *Journal of Biological Chemistry*.

[71] Barreau C, Paillard L, Osborne HB (2005). AU-rich elements and associated factors: are there unifying principles?. *Nucleic Acids Research*.

[72] Brennan CM, Steitz JA (2001). HuR and mRNA stability. *Cellular and Molecular Life Sciences*.

[73] Tulis DA, Durante W, Liu X, Evans AJ, Peyton KJ, Schafer AI (2001). Adenovirus-mediated heme oxygenase-1 gene delivery inhibits injury-induced vascular neointima formation. *Circulation*.

[74] Morita T (2005). Heme oxygenase and atherosclerosis. *Arteriosclerosis, Thrombosis, and Vascular Biology*.

[75] Durante W (2003). Heme oxygenase-1 in growth control and its clinical application to vascular disease. *Journal of Cellular Physiology*.

[76] Lee T-S, Chau L-Y (2002). Heme oxygenase-1 mediates the anti-inflammatory effect of interleukin-10 in mice. *Nature Medicine*.

[77] Ellison S, Gabunia K, Kelemen S, Scalia R, Autieri M (2012). Attenuation of experimental atherosclerosis by systemic administration of
interleukin-19. *Arteriosclerosis, Thrombosis and Vascular Biology*.

[78] Chen W-Y, Cheng BC, Jiang MJ, Hsieh MY, Chang MS (2006). IL-20 is expressed in atherosclerosis plaques and promotes atherosclerosis in apolipoprotein E-deficient mice. *Arteriosclerosis, Thrombosis, and Vascular Biology*.

[79] Schulte S, Sukhova GK, Libby P (2008). Genetically programmed biases in Th1 and Th2 immune responses modulate atherogenesis. *American Journal of Pathology*.

[80] Liu W, Li WM, Gao C, Sun NL (2005). Effects of atorvastatin on the Th1/Th2 polarization of ongoing experimental autoimmune myocarditis in Lewis rats. *Journal of Autoimmunity*.

